# Kinetic Studies on Fermentative Production of Biofuel from Synthesis Gas Using *Clostridium ljungdahlii*

**DOI:** 10.1155/2014/910590

**Published:** 2014-01-30

**Authors:** Maedeh Mohammadi, Abdul Rahman Mohamed, Ghasem D. Najafpour, Habibollah Younesi, Mohamad Hekarl Uzir

**Affiliations:** ^1^Faculty of Chemical Engineering, Babol Noushirvani University of Technology, Babol 47148, Iran; ^2^Low Carbon Economy (LCE) Research Group, School of Chemical Engineering, Engineering Campus, Universiti Sains Malaysia, 14300 Nibong Tebal, Penang, Malaysia; ^3^Department of Environmental Science, Faculty of Natural Resources and Marine Science, Tarbiat Modares University, Nour 46414, Iran

## Abstract

The intrinsic growth, substrate uptake, and product formation biokinetic parameters were obtained for the anaerobic bacterium, *Clostridium ljungdahlii*, grown on synthesis gas in various pressurized batch bioreactors. A dual-substrate growth kinetic model using Luong for CO and Monod for H_2_ was used to describe the growth kinetics of the bacterium on these substrates. The maximum specific growth rate (*μ*_max_ = 0.195 h^−1^) and Monod constants for CO (*K*_*s*,CO_ = 0.855 atm) and H_2_ (*K*_*s*,H_2__ = 0.412 atm) were obtained. This model also accommodated the CO inhibitory effects on cell growth at high CO partial pressures, where no growth was apparent at high dissolved CO tensions (*P*_CO_^∗^ > 0.743 atm). The Volterra model, Andrews, and modified Gompertz were, respectively, adopted to describe the cell growth, substrate uptake rate, and product formation. The maximum specific CO uptake rate (*q*_max_ = 34.364 mmol/g_cell_/h), CO inhibition constant (*K*_*I*_ = 0.601 atm), and maximum rate of ethanol (*R*_max_ = 0.172 mmol/L/h at *P*_CO_ = 0.598 atm) and acetate (*R*_max_ = 0.096 mmol/L/h at *P*_CO_ = 0.539 atm) production were determined from the applied models.

## 1. Introduction

The production of fuels and high value chemicals from synthesis gas has become an intriguing target, since the beginning of the 20th century. However, most of the developments and explorations in this area have been concentrated on the deployment of metal-based catalytic processes. Recently, attention has been turned towards the conversion of synthesis gas to biofuels and biochemicals through the microbial routes due to the advantages offered by microbes as biocatalysts over metal-based catalysts [1]. Fermentation of synthesis gas to produce second-generation biofuels would likely be an option to address part of the debates over production of fuels from food crops. Despite recent research and endeavors, fermentation of synthesis gas to biofuels is still a relatively immature technology to be demonstrated at a commercial scale and various technical and economical challenges should be obviated for its future commercial deployments [2].

*Clostridium ljungdahlii* is a strictly anaerobic acetogen known for its ability to grow autotrophically on synthesis gas constituents and ferment them to ethanol and acetate [3]. The bacterium catalyzes a Fischer-Tropsch type reaction under a mild condition of pressure and temperature to produce fuel and chemical. The overall pathway reaction which forms acetate from CO as well as H_2_/CO_2_ has been established for many acetogens [4]:

(1) $$\begin{array}{c} 4\text{CO}+2{\text{H}}_{2}\text{O}⟶{\text{CH}}_{3}\text{COOH}+2{\text{CO}}_{2} \end{array}$$

(2) $$\begin{array}{c} 2{\text{CO}}_{2}+4{\text{H}}_{2}⟶{\text{CH}}_{3}\text{COOH}+2{\text{H}}_{2}\text{O} \end{array}$$

The stoichiometries for ethanol formation have not been determined due to the lack of autotrophic ethanologenic organisms. However, from a similarity point of view with respect to the reactions established for acetate formation, the following reactions have been proposed [4]:

(3) $$\begin{array}{c} 6\text{CO}+3{\text{H}}_{2}\text{O}⟶{\text{C}}_{2}{\text{H}}_{5}\text{OH}+4{\text{CO}}_{2} \end{array}$$

(4) $$\begin{array}{c} 2{\text{CO}}_{2}+6{\text{H}}_{2}⟶{\text{C}}_{2}{\text{H}}_{5}\text{OH}+3{\text{H}}_{2}\text{O} \end{array}$$

In the above reactions, the reducing equivalents are served by CO and H_2_ and the stoichiometric coefficients are identical for these reduced species [5].

The aim of this study was to determine the intrinsic reaction and biokinetic parameters for *C. ljungdahlii* grown on CO and H_2_ at various initial gas pressures. It is well understood that the microbial growth and product distribution of microorganism during gas fermentation can be significantly affected by the partial pressure of the gas components, as the enzyme involved in the metabolic pathway of the organism is sensitive to substrate exposure [6]. In most studies conducted so far, the composition of gas was selected in a way that only one component was the dominant substrate [4, 5, 7, 8]. In those cases, a single substrate growth model is utilized to determine the biokinetic parameters. Phillips et al. [5] grew *C. ljungdahlii* in batch cultures with H_2_/CO_2_ or CO/CO_2_ as the growth substrates to assess the influence of substrate on fermentation parameters used in process design. They concluded that CO provided a higher specific growth rate (*μ* = 0.06 h^−1^), whereas H_2_ brought about a higher specific uptake rate (*q* = 0.079 mol/g_cell_/h). Vega et al. [9] obtained the fermentation parameters of *Peptostreptococcus productus* on gaseous substrates (CO, H_2_/CO_2_). Based on the achieved results it was deduced that only CO was consumed for the growth, whereas CO and CO_2_/H_2_ were utilized for production of acetate. They used some kinetic models including substrate inhibition to determine the biokinetic parameters; the results were *μ*_max⁡_ = 0.21 h^−1^ and *q*_max⁡_ = 144 mmol/g_cell_/h with CO inhibitory effects on cell growth (*P*_CO_^*L*^ > 60 kPa) and gas uptake (*P*_CO_^*L*^ > 80 kPa).

In this work, the composition of synthesis gas was defined so that the partial pressures of CO, H_2_, and CO_2_ were the same, which simulates the composition of synthesis gas obtained from the air-catalytic gasification [10]. In this case, the microbial growth is affected by more than a single substrate. In order to take into account such possibility, a dual-substrate growth model was used for *C. ljungdahlii* and the growth kinetic parameters were determined. Substrate inhibition effects in relation to the dissolved CO tension were discussed. Substrate uptake rate, mass transfer kinetics, and kinetics of product formation were also aspects of the discussion.

## 2. Materials and Methods

### 2.1. Microorganism and Growth Medium

*Clostridium ljungdahlii* (ATCC 55383) was grown anaerobically in a medium containing mineral salts, vitamins, and trace metals from ATCC 1754 PETC. The basal medium contained (per 1.0 L) mineral salts (NH_4_Cl (1.0 g), KCl (0.1 g), MgSO_4_·7H_2_O (0.2 g), NaCl (0.8 g), KH_2_PO_4_ (0.1 g), and CaCl_2_·2H_2_O (20.0 mg)), 1.0 g yeast extract, 10 mL trace elements solution, 10 mL vitamins solution, and 10 mL reducing agent. The trace elements solution contained (per 1.0 L) nitrilotriacetic acid (2.0 g), MnSO_4_·H_2_O (1.0 g), Fe (SO_4_)_2_ (NH_4_)_2_·6H_2_O (0.8 g), CoCl_2_·6H_2_O (0.2 g), ZnSO_4_·7H_2_O (0.2 mg), CuCl_2_·2H_2_O (20.0 mg), NiCl_2_·6H_2_O (20.0 mg), Na_2_MoO_4_·2H_2_O (20.0 mg), Na_2_SeO_4_ (20.0 mg), and Na_2_WO_4_ (20.0 mg). The vitamins solution contained (per 1.0 L) biotin (2.0 mg), folic acid (2.0 mg), pyridoxine hydrochloride (10.0 mg), thiamine-HCl (5.0 mg), riboflavin (5.0 mg), nicotinic acid (5.0 mg), calcium D-(+)-pantothenate (5.0 mg), vitamin B12 (0.1 mg), p-aminobenzoic acid (5.0 mg), and thioctic acid (5.0 mg). The reducing agent solution contained (per 100 mL) NaOH (0.9 g), L-Cysteine-HCl (4.0 g), and Na_2_S·9H_2_O (4.0 g).

### 2.2. Batch Fermentation Experiments

The medium (excluding the reducing agent) was prepared, boiled, and dispensed anaerobically under nitrogen atmosphere into several Wheaton serum bottles (Borosilicate glass, Fischer Scientific, UK). Each serum bottle (163 mL) was filled with 50 mL of liquid medium. The reducing agent solution was prepared in separate serum bottle. All bottles were autoclaved at 121°C for 20 min. The cool sterilized medium in each serum bottle was reduced by the addition of 1.6 mL reducing agent (per 50 mL medium). The pH of the media was adjusted to 5.9 using 1 M HCl or NaOH; then the synthesis gas containing CO, CO_2_, H_2_, and Ar (30, 30, 30, and 10%, resp.) was purged into the bottles. Argon was used as internal standard to determine the total pressure changes inside the bottles and this inert gas did not interfere with the ability of the bacterium to produce ethanol and acetate. The bottles were then flushed with the substrate gas and pressurized to various initial pressures of 0.2, 0.5, 0.8, 1.0, 1.2, and 1.5 atm (gauge). The media were inoculated (10% v/v) with seed culture harvested from a fermenter (Infors, Switzerland), operating with a continuous flow of synthesis gas (the same composition as the substrate gas in bottles) and medium defined. The bottles were then placed horizontally in an incubator shaker at 37°C and 150 rpm. For determination of the gas composition, optical density and ethanol and acetate concentrations samples were taken periodically at appropriate intervals.

### 2.3. Cell Density and Product Analysis

Optical density of the samples was analyzed using a spectrophotometer (Thermo Spectronic, USA) at 580 nm and the cell dry weight was determined using a predeveloped calibration curve. In order to calculate the substrate gas consumption, the gas phase was analyzed using a gas chromatograph (Perkin Elmer, Autosystem XL), equipped with a thermal conductivity detector (TCD) and a packed column, 15 ft × 1/8 in., 100/120 mesh, Carboxene 1000 (Supelco). Detection of ethanol and acetate was performed using another gas chromatograph (Agilent, 5890 series II), equipped with a flame ionization detector (FID). The chromatography column was 80/120 mesh Carbopack B-DA/4% Carbowax 20 M (Supelco, USA). The samples were previously acidified and 2-pentanone was used as internal standard.

## 3. Results and Discussion

### 3.1. Kinetics of Microbial Growth

In batch cultivation of *C. ljungdahlii*, the cells started the exponential growth without going through any lag phase. This was because of the use of metabolically active inocula, preadapted to the synthesis gas, which were harvested from an actively operating bioreactor. Almost all cultures, with various initial gas pressures, reached a maximum growth after which the culture density decreased. To describe the growth profile of *C. ljungdahlii *on synthesis gas at various initial pressures, Volterra kinetic model was used. This model is able to predict both the birth and death phases of growth and considers them the only factors which change the cell population in a batch system [7, 11]:

(5) $$\begin{array}{c} x=\frac{x_{0}\exp\left(\mu_{m}t\right)}{1-{\left(x_{0}/x_{m}\right)}^{2}\left(\mu_{m}/\left(k+\mu_{m}\right)\right)\left(1-\exp\left(\left(k+\mu_{m}\right)\cdot t\right)\right)}, \end{array}$$

where *x*_0_ is the initial cell concentration (g/L), *x*_*m*_ expresses the maximum cell density (g/L), *μ*_*m*_ is the maximum specific growth rate (h^−1^), *t* represents the fermentation period (h), and *k* is the cell decline or promotion constant (h^−1^).

**Figure 1:**
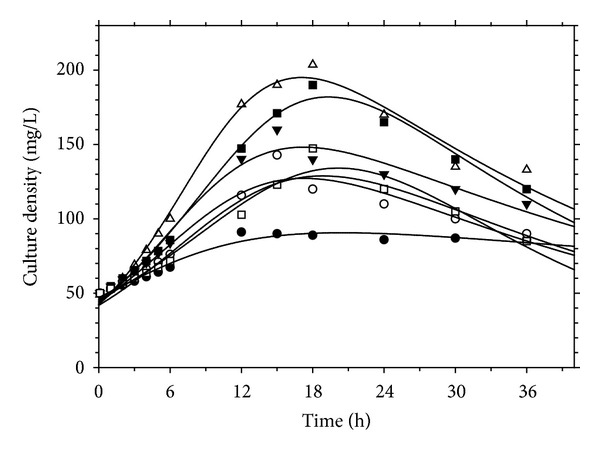
The Volterra model applied to the experimental data at various synthesis gas pressures; ●: 0.2, ○: 0.5, *▼*: 0.8, Δ: 1.0, ■: 1.2, □: 1.5 (atm), and —: (5).

Figure 1 shows the Volterra model applied to the experimental data. The model fitted to the experimental results with high regression coefficients (*R* ^2^) at all specified pressures. The kinetic parameters of this model are summarized in Table 1.

The constant *k* is associated with the inhibition or promotion of the cell growth. A negative value of *k* implies that the cell growth is promoted, whereas a positive value is an indication of inhibition caused by toxic chemicals. A positive value of *k* was achieved in all cases indicating that the bacterial cell growth declined after reaching a maximum cell concentration due to some inhibitory effects caused by CO. Generally, in biological processes, reactant products or contaminants can impose inhibitory effect on cell growth and/or product formation. In the case of synthesis gas fermentation, CO could be the most probable potential source of inhibition. CO_2_ can also be another potential source of inhibition, as it affects the medium pH by the formation of carbonic acid or its carbonate derivatives [12]. However, in this experiment, no appreciable pH drop occurred in the cultures, implying that CO_2_ did not cause inhibition. Increase of H_2_ partial pressure in the gas phase or its accumulation in the fermentation medium can also impose inhibition on the fermentation process due to the alteration of carbon flow in the biological pathway of microorganism [13]. Nevertheless, the solubility of H_2_ in liquid medium is much less than that of CO [14]; thus, it was speculated that CO was the primary source of inhibition in the current experiment.

As observed in Figure 1, the cell population declined, after reaching a maximum, without experiencing any stationary phase. It could be deduced that the cell population in the batch bioreactors was not enough to consume all the CO transferred to the liquid phase. This probably resulted in the buildup of liquid phase concentration of CO which eventually inhibited the cell growth and caused the cell death. Such inhibitory effect was more severe at high synthesis gas pressures. Although the higher concentration gradients increased the mass transfer rate, this promoted rate was somewhat higher than the rate of CO uptake which caused inhibition on growth rate.

According to Bailey and Ollis [15], when microbial cell growth is limited by more than one substrate, three forms of multiple-substrate growth models need to be considered to describe the growth.

Interactive or multipleactive form is

(6) $$\begin{array}{c} \frac{\mu }{\mu_{\max}}=\left[\mu \left(S_{1}\right)\right]\left[\mu \left(S_{2}\right)\right]\cdots \left[\mu \left(S_{i}\right)\right]. \end{array}$$

Additive form is

(7) $$\begin{array}{c} \frac{\mu }{\mu_{\max}}=\frac{\left[\mu \left(S_{1}\right)+\mu \left(S_{2}\right)+\cdots +\mu \left(S_{i}\right)\right]}{i}. \end{array}$$

Noninteractive form is

(8) $$\begin{array}{c} \frac{\mu }{\mu_{\max}}=\mu \left(S_{1}\right)\;\text{or}\;\mu \left(S_{2}\right)\;\text{or}\cdots \text{or}\;\mu \left(S_{i}\right). \end{array}$$

It was hypothesized that *C. ljungdahlii* was able to grow on either of the limiting substrates, hydrogen or carbon monoxide, based on reactions (1)–(4), and the microbial growth was not hindered in the absence of any of them. To prove this, a plot of cell concentration versus the consumption of both substrates (CO and H_2_) was developed and a linear relationship was found indicating that both substrates were consumed for cell growth (data not shown) [9]. This implies that the growth kinetic followed the additive form (7), and (6) and (8) were not applicable in this case.

In order to describe the microbial growth rate (*μ*) as a function of dual-substrate (CO, H_2_) concentrations, various single substrate growth models (Table 2), including substrate inhibition models, were combined as described by (7).

Various combinations of several kinetic models (presented in Table 3) in additive form were used to develop the biokinetic expressions able to describe the growth behavior of *C. ljungdahlii*. The sum of squares of differences (SSD) between the experimentally measured specific growth rates and the calculated ones by the mathematical models was used as a predefined objective function:

(9) $$\begin{array}{c} \text{SSD}=\overset{N}{\underset{i=1}{\sum }}{\left(\mu_{\exp}-\mu_{\text{model}}\right)}^{2}. \end{array}$$

Minimizing this objective function was required to calculate the biokinetic parameters from the experimental data [16]. To improve the accuracy of the double parametric model, random data sets, similar to the experimental data, within the range of dissolved CO and H_2_ tensions and calculated specific growth rates, were generated using standard Excel RAND function. The generated data were used to evaluate the suitability of the developed kinetic models to predict the dual-substrate growth rate of *C. ljungdahlii*. Sigma Plot 11 was used to develop the combined kinetic models and to calculate the biokinetic parameters. Models that resulted in negative values extremely high or out of range biokinetic constants were rejected. The best dual-substrate growth model was selected among the different additive combinations of the models presented in Table 2.

**Figure 2:**
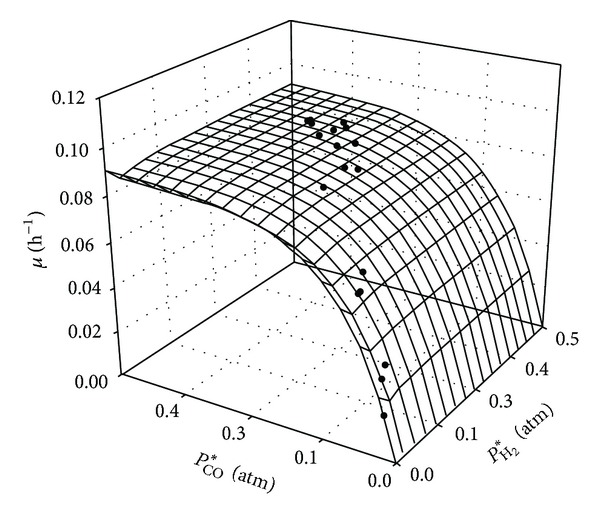
The specific growth rate predicted from (10) applied to the experimental data.

The calculated biokinetic parameters and SSD values for each combined model are tabulated in Table 3. The minimum SSD was found for an additive combination of Luong and Monod kinetics. This model was selected as the best kinetic model to describe the growth of *C. ljungdahlii* on CO and H_2_:

(10) μ=μmax⁡  2((SCO/(Ks,CO+SCO))(1−SCO/Sm)n+SH2/(Ks,H2+SH2)),

where *μ*_max⁡  _ is the maximum specific growth rate (h^−1^), *K*_*s*,CO_ and *K*_*s*,H_2__ are the Monod constant for CO and H_2_ (atm), *S*_*m*,CO_ represents the maximum inhibitory concentration of CO at which no growth is apparent, and *n* is the constant for Luong model which accounts for the relationship between *μ* and *S* [17]. For the selected model, the biokinetic parameters were obtained as *μ*_max⁡_ = 0.195 h^−1^, *K*_*s*,CO_ = 0.855 atm, *K*_*s*,H_2__ = 0.412 atm, *S*_*m*,CO_ = 0.743 atm, and *n* = 0.465.  Figure 2 shows the specific growth rate as a function of the CO and H_2_ dissolved tensions; the experimental data were fitted to (10).

### 3.2. Kinetics of Substrate Gas Utilization

The anaerobic bacterium *C. ljungdahlii* is able to utilize CO through the Wood-Ljungdahl pathway, to produce acetate and ethanol based on (1) and (3). An alternative route involves the conversion of CO_2_ and H_2_ to ethanol and acetate through the same set of enzymes and transformation mechanism encompassed by the acetyl-CoA pathway according to (2) and (4). In the current study, H_2_ consumption was higher than CO at all gas pressures. Such observation signifies that the bacteria consumed H_2_/CO_2_ as the preferred substrate through the homoacetic pathway. The highest substrate conversion efficiency of 76% for H_2_ and 67% for CO was achieved at the initial synthesis gas pressure of 1.0 atm.

To describe the substrate gas utilization by the bacterium, a first order reaction kinetic was used. The expression for CO consumption with respect to the fermentation time in the form of first order differential equation is as follows:

(11) $$\begin{array}{c} -\frac{dP_{\text{CO}}^{*}}{dt}=\;k_{p}P_{\text{CO}}^{*}. \end{array}$$

The expression for time dependent CO liquid phase concentration is obtained by integrating from the first order consumption rate which leads to:

(12) $$\begin{array}{c} P_{\text{CO}}^{*}=P_{\text{CO},0}^{*}\exp\left(k_{p}t\right), \end{array}$$

where *P*_CO,0_* and *P*_CO_* are the initial and instantaneous dissolved CO tensions in the liquid phase (atm), *k*_*p*_ is the first order rate constant (h^−1^), and *t* is the fermentation time (h).

**Figure 3:**
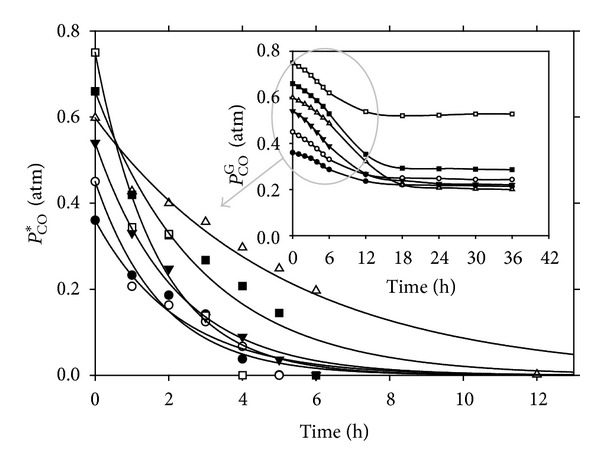
Measured CO partial pressure in the gas phase (the inset) and calculated dissolved CO tension in liquid in various pressurized batch bioreactors; ●: 0.2, ○: 0.5, *▼*: 0.8, Δ: 1.0, ■: 1.2, and □: 1.5 (atm).

Figure 3 describes the dissolved CO reduction in batch culture for various pressurized bioreactors. As observed, the first order reaction model was suitable to describe the kinetics of the CO utilization with high regression coefficient (*R* ^2^ > 0.94) for all pressures. However, study of the effect of initial gas pressure on substrate gas uptake showed the potential inhibitory effect of CO on the cell growth and substrate gas uptake at pressures above 1.0 atm. Increase of the initial gas pressure from 0.2 to 1.0 atm improved the substrate gas utilization due to the enhanced mass transfer rates. However, increase of the gas pressure to beyond 1.0 atm adversely affected the substrate gas uptake and cell growth. At high pressures, the dissolved CO concentration reached toxic levels, as the cell concentration was insufficient to keep the reaction in mass transfer limited regime. The increase of the reactor pressure (above 1.0 atm) improved the mass transfer rate above the intrinsic biological reaction rate. As a result, buildup of the liquid phase concentration of CO imposed an inhibitory effect on the microbial growth and gas uptake. These results suggest the employment of pressurized operation as long as the cell concentration is high enough to keep the dissolved CO tensions at low levels to prevent any possibility of CO inhibition.

### 3.3. Kinetics of Substrate Gas Uptake Rate and Mass Transfer

The data obtained at various gas pressures were used to determine the kinetics of CO uptake rate using a modified Monod model proposed by Andrews which accommodates CO inhibition [18]:

(13) $$\begin{array}{c} q_{\text{CO}}=\frac{q_{\max}P_{\text{CO}}^{*}}{K_{p}+P_{\text{CO}}^{*}+{P_{\text{CO}}^{*}}^{2}/K_{I}}, \end{array}$$

where *P*_CO_* is the dissolved CO tension in the liquid phase (atm), *q*_max⁡_ represents the maximum specific uptake rate (mmol/g_cell_/h), *K*_*p*_ is the Monod constant (atm), and *K*_*I*_ is the CO inhibition constant (atm).

Determination of the kinetic parameters in (13) is not straightforward due to the difficulties involved in measuring the liquid phase concentration of CO. The procedure to find the dissolved CO tension involves the determination of the mass transfer coefficient (*K*_L_ *a*) using a mass balance on the rate of transfer of CO to the liquid medium:

(14) $$\begin{array}{c} -\frac{1}{V_{\text{L}}}\frac{dn_{\text{CO}}^{\text{G}}}{dt}=\frac{K_{\text{L}}a}{H}\left(P_{\text{CO}}^{\text{G}}-P_{\text{CO}}^{*}\right), \end{array}$$

where *V*_L_ is the medium volume (L), *n*_CO_^G^ represents the moles of CO in the gas phase (mmol), *t* is the fermentation time (h), *K*_L_ *a* corresponds to the mass transfer coefficient (h^−1^), *H* is the Henry's constant (atm·L/mmol), and *P*_CO_^G^ and *P*_CO_* represent the gas phase and dissolved CO tension in the liquid phase (atm), respectively. Under the mass transfer controlled condition, where the *P*_CO_* is assumed to be zero, *K*_L_ *a* is obtained from the following equation:

(15) $$\begin{array}{c} -\frac{1}{V_{\text{L}}}\frac{dn_{\text{CO}}^{\text{G}}}{dt}=\frac{K_{\text{L}}a}{H}P_{\text{CO}}^{\text{G}}. \end{array}$$

The value of *P*_CO_* is calculated at the early stage of the fermentation process, where the mass transfer is not the controlling mechanism.

The rate of disappearance of CO in the liquid phase as a result of microbial uptake is obtained by the following equation:

(16) $$\begin{array}{c} q_{\text{CO}}=\frac{1}{xV_{\text{L}}}\frac{dn_{\text{CO}}}{dt}, \end{array}$$

where *q*_CO_ represents the specific uptake rate (mmol/g_cell_/h) and *x* is the cell density (g/L). By rearranging (13) to form (17), the kinetic parameters are found by a linear and quadratic regression:

(17) $$\begin{array}{c} \frac{P_{\text{CO}}^{*}}{q_{\text{CO}}}=\frac{K_{P}}{q_{\max}}+\frac{P_{\text{CO}}^{*}}{q_{\max}}+\frac{{P_{\text{CO}}^{*}}^{2}}{q_{\max}K_{I}}. \end{array}$$

**Figure 4:**
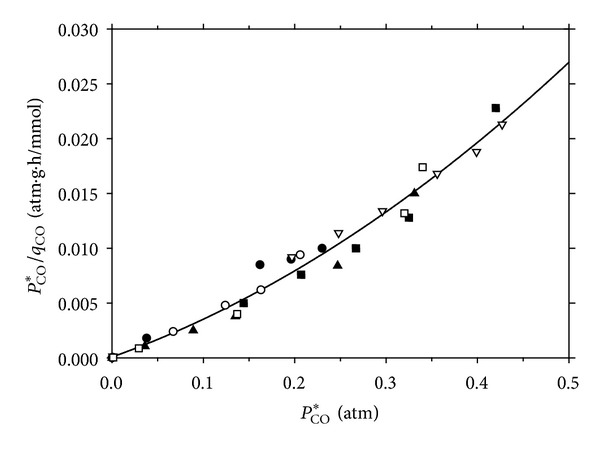
Linear and quadratic Andrews model for CO uptake by *C. ljungdahlii* at various gas pressures; ●: 0.2, ○: 0.5, *▼*: 0.8, Δ: 1.0, ■: 1.2, and □: 1.5 (atm).

Figure 4 shows the specific CO uptake rate for various initial gas pressures. As observed, all data were represented by a single quadratic curve with a high regression coefficient (*R* ^2^ = 0.97). The final expression correlating the specific CO uptake rate of *C. ljungdahlii* is described by the following equation:

(18) $$\begin{array}{c} q_{\text{CO}}=\frac{34.36P_{\text{CO}}^{*}}{0.021+P_{\text{CO}}^{*}+{P_{\text{CO}}^{*}}^{2}/0.601}. \end{array}$$

The maximum specific CO uptake rate, *q*_max⁡_, was obtained as 34.364 mmol/g_cell_/h and the Monod constant, *K*_*p*_, was 0.021 atm. The CO inhibition constant, *K*_*I*_, had a value of 0.601 atm indicating that the dissolved CO significantly inhibits CO uptake.

### 3.4. Kinetics of Product Formation

*C. ljungdahlii* as an ethanologenic acetogen presents a complex metabolic pathway which includes both acetogenic and solventogenic phases [19]. Although ethanol is the desirable product from the solventogenic fermentation of synthesis gas, acetate is the main product formed through the acetogenic pathway. The formation kinetics of ethanol or acetate as the fermentation products was studied using a modified Gompertz equation which is defined as [20]

(19) $$\begin{array}{c} P=P_{\max}\exp\left(-\exp\left[\frac{R_{\max}\times e}{P_{\max}}\left(\lambda -t\right)+1\right]\right), \end{array}$$

where *P* represents the amount of product (ethanol or acetate) formed (mmol/L), *P*_max⁡_ is the maximum product formed (mmol/L), *R*_max⁡_ is the maximum rate of production (mmol/L/h), *λ* is the lag time to exponential product formation (h), and *t* is the fermentation time (h).

**Figure 5:**
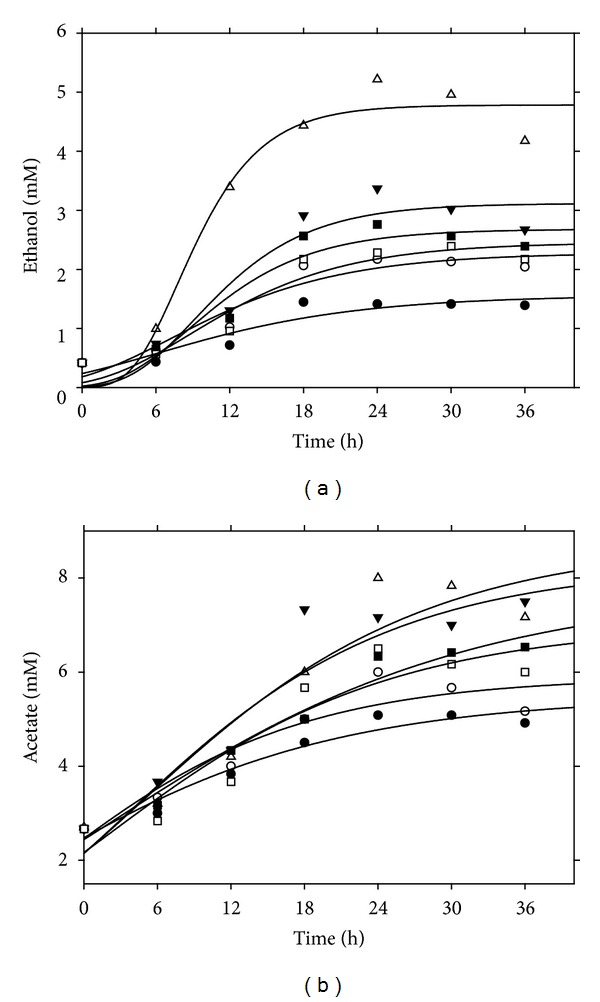
Modified Gompertz model for the formation of (a) ethanol and (b) acetate at various gas pressures; ●: 0.2, ○: 0.5, *▼*: 0.8, Δ: 1.0, ■: 1.2, and □: 1.5 (atm).

The fitted curves for ethanol and acetate are depicted in Figures 5(a) and 5(b) and the kinetic parameters for various pressurized bioreactors are tabulated in Table 4. The correlation coefficients were almost above 0.90 in all cases suggesting the suitability of the modified Gompertz model to describe the formation of ethanol and acetate by *C. ljungdahlii* during the batch fermentation of synthesis gas.

## 4. Conclusion

Fermentation parameters of *C. ljungdahlii* grown on synthesis gas at various initial gas pressures were obtained using several unstructured kinetic models. A dual-substrate growth model which combined Luong (for CO) and Monod (for H_2_) kinetics was used to describe the growth rate of the bacterium on CO and H_2_. Volterra kinetic model and Andrews which both include possible inhibitions were applied to describe the cell growth and CO uptake rate. The kinetic models confirmed possible CO inhibition on cell growth and gas uptake at high dissolved CO tensions which were experimentally observed in pressurized batch bioreactors. The modified Gompertz equation employed to model the product formation was also suitable to describe the ethanol and acetate formation.

## Figures and Tables

**Table 1:** Kinetic parameters based on Volterra model for growth of *C. ljungdahlii*.

Initial gas pressure atm	*x*_0_ mg/L	*x*_*m*_ mg/L	*μ*_*m*_ h^−1^	*k* h^−1^	*R* ^2^
0.2	47.07	70.79	0.153	0.008	0.972
0.5	43.92	97.51	0.155	0.031	0.947
0.8	42.80	98.94	0.185	0.025	0.969
1.0	44.48	150.89	0.196	0.033	0.987
1.2	44.76	135.63	0.145	0.042	0.991
1.5	41.87	101.00	0.138	0.034	0.948

**Table 2:** Various single substrate kinetic models used to develop a dual-substrate growth model.

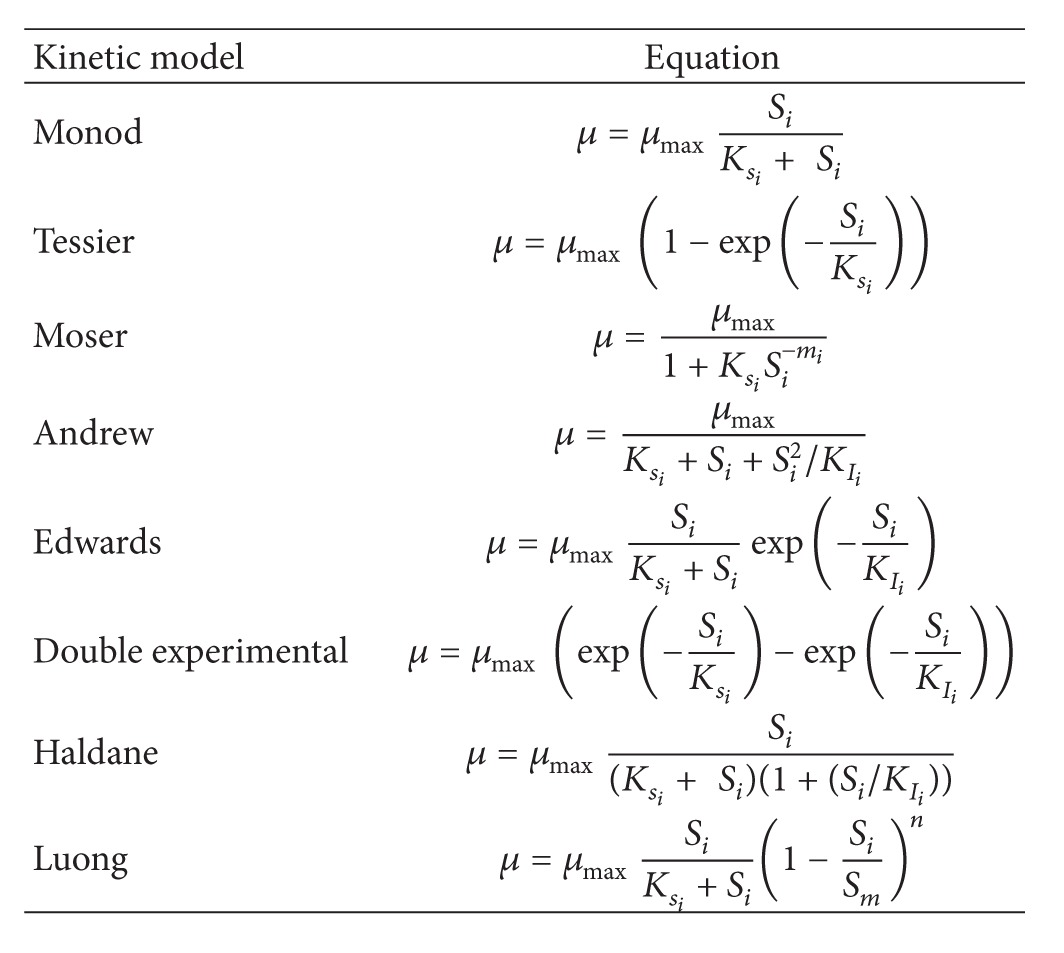

**Table 3:** The developed dual-substrate growth models to describe the growth kinetics of *C. ljungdahlii* on H_2_ and CO, biokinetic parameters, and SSD.

Model*	*µ*_max⁡_ h^−1^	*K*_*s*,CO_ atm	*K*_*S*,H_2__ atm	*K*_*I*,CO_ atm	*S*_*m*,CO_ atm	*n* —	SSD
Luong + Monod	0.1951	0.8551	0.4124	—	0.7432	0.4652	0.0005
Double-experimental + Monod	0.1763	0.3051	0.3006	0.3741	—	—	0.0010
Luong + Tessier	0.1600	0.8524	0.4120	—	0.7431	0.4736	0.0010
Double-experimental + Moser	0.1764	0.3051	0.3008	0.3740	—	—	0.0010
Luong + Moser	0.1009	0.3070	0.3001	—	0.7470	0.1249	0.0011
Double-experimental + Tessier	0.1473	0.6353	0.3990	0.4713	—	—	0.0013
Andrew + Tessier	0.1555	1.038	0.3290	0.4001	—	—	0.0014
Edwards + Monod	0.1755	0.6396	0.3912	0.6998	—	—	0.0014
Edwards + Tessier	0.1585	0.8110	0.399	0.6243	—	—	0.0014
Andrew + Monod	0.1650	0.6995	0.3778	0.4876	—	—	0.0015
Haldane + Moser	0.1580	0.5393	0.4050	0.7315	—	—	0.0015
Haldane + Monod	0.1549	0.5573	0.3749	0.7128	—	—	0.0016

*In each combination, the first model is used for CO and the second one for H_2_.

**Table 4:** Calculated biokinetic parameters using the modified Gompertz model for product formation.

Product	Pressure atm	*P*_max⁡_ mmol/L	*R*_max⁡_ mmol/L/h	*λ* h	*R* ^2^
Ethanol	0.2	1.5742	0.0223	6.0333	0.9125
	0.5	2.2386	0.0394	7.3126	0.9193
	0.8	3.1463	0.0808	9.1129	0.8989
	1.0	4.7592	0.1722	7.7737	0.9624
	1.2	2.5861	0.0672	8.6630	0.9073
	1.5	1.9433	0.0464	8.7915	0.9153

Acetate	0.2	5.0161	0.0542	3.1672	0.9509
	0.5	5.1703	0.0706	2.5373	0.9050
	0.8	6.9560	0.0963	2.3957	0.9053
	1.0	8.2264	0.0877	4.6254	0.9037
	1.2	5.8639	0.0618	2.5416	0.9708
	1.5	5.5812	0.0711	2.1636	0.8928

## Nomenclature

*H*: Henry's constant (atm·L/mmol)
*k*: Cell decline or promotion constant (h^−1^)
*K*_*I*,CO_: Inhibition constant for CO (atm)
*K*_L_*a*: Mass transfer coefficient (h^−1^)
*k*_*p*_: First order rate constant (h^−1^)
*K*_*s*,CO_: Monod half-saturation constant for CO (atm)
*K*_*s*,H_2__: Monod half-saturation constant for H_2_ (atm)
*m*: Moser's constant for substrate *i* (g/L)
*n*: Constant for Loung model
*n*_CO_^G^: Moles of CO in the gas phase (mmol)
*P*_CO_*: Instantaneous dissolved CO tension in the liquid phase (atm)
*P*_CO,0_*: Initial dissolved CO tension in the liquid phase (atm)
*P*_CO_^G^: Gas phase pressure of CO (atm)
*P*: Amount of product formed (mmol/L)
*P*_max⁡_: Maximum product formed (mmol/L)
*q*_CO_: Specific CO uptake rate (mmol/g_cell_/h)
*q*_max⁡_: Maximum specific uptake rate (mmol/g_cell_/h)
*R*_max⁡_: Maximum rate of production (mmol/L/h)
*S*_*i*_: Pressure of substrate *i* (atm)
*S*_*m*,CO_: Maximum inhibitory pressure of CO at which no growth is apparent (atm)
SSD: Sum of squares of differences
*t*: Fermentation period (h)
*V*_L_: Volume of medium (L)
*x*: Instantaneous cell concentration (g/L)
*x*_0_: Initial cell concentration (g/L)
*x*_*m*_: Maximum cell concentration (g/L).

### Greek Symbols

*μ*: Specific growth rate (h^−1^)
*μ*_*m*_: Maximum specific growth rate (h^−1^)
*μ*_exp⁡_: Experimentally determined specific growth rate (h^−1^)
*μ*_model_: Specific growth rate predicted from model (h^−1^)
*λ*: Lag time to exponential product formation (h).
